# Comprehensive Analysis of Calcium Sensor Families, CBL and CIPK, in *Aeluropus littoralis* and Their Expression Profile in Response to Salinity

**DOI:** 10.3390/genes14030753

**Published:** 2023-03-20

**Authors:** Mozhdeh Arab, Hamid Najafi Zarrini, Ghorbanali Nematzadeh, Parviz Heidari, Seyyed Hamidreza Hashemipetroudi, Markus Kuhlmann

**Affiliations:** 1 Department of Plant Biotechnology, Sari Agricultural Sciences and Natural Resources University (SANRU), Sari 4818166996, Irannajafi316@yahoo.com (H.N.Z.);; 2 National Institute of Genetic Engineering and Biotechnology (NIGEB), Tehran 14965161, Iran; 3 Department of Genetic Engineering and Biology, Genetics and Agricultural Biotechnology Institute of Tabarestan (GABIT), Sari Agricultural Sciences and Natural Resources University (SANRU), Sari 4818166996, Iran; 4 Faculty of Agriculture, Shahrood University of Technology, Shahrood 3619995161, Iran; 5 RG Heterosis, Leibniz Institute of Plant Genetics and Crop Plant Research (IPK), 306466 Gatersleben, Germany

**Keywords:** calcium sensors, CBL, CIPK, salt stress, kinases, cell signaling

## Abstract

Plants have acquired sets of highly regulated and complex signaling pathways to respond to unfavorable environmental conditions during evolution. Calcium signaling, as a vital mechanism, enables plants to respond to external stimuli, including abiotic and biotic stresses, and coordinate the basic processes of growth and development. In the present study, two calcium sensor families, CBL and CIPK, were investigated in a halophyte plant, *Aeluropus littoralis*, with a comprehensive analysis. Here, six *AlCBL* genes, and twenty *AlCIPK* genes were studied. The analysis of the gene structure and conserved motifs, as well as physicochemical properties, showed that these genes are highly conserved during evolution. The expression levels of *AlCBL* genes and *AlCIPK* genes were evaluated under salt stress in leaf and root tissue. Based on the real-time RT-PCR results, the *AlCIPK* gene family had a higher variation in mRNA abundance than the *AlCBL* gene family. *AlCIPK* genes were found to have a higher abundance in leaves than in roots. The results suggest that the correlation between *AlCBL* genes and *AlCIPK* is tissue-specific, and different correlations can be expected in leaves and roots. Based on these correlations, AlCIPK3.1–AlCBL4.1 and AlCIPK1.2–AlCBL4.4 can be co-expressed in the root tissue, while AlCBL10 has the potential to be co-expressed with AlCIPK5, AlCIPK26, and AlCIPK12.3 in the leaf tissue. Our findings reveal valuable information on the structure and function of calcium sensor families in *A. littoralis,* a halophyte plant, that can be used in future research on the biological function of CBLs and CIPKs on salt stress resistance.

## 1. Introduction

In sessile organisms such as plants, perception and signaling of environmental stimuli are necessary for survival and growth regulation. Calcium (Ca^2+^) is one of the signal transduction components that acts as a second messenger in all eukaryotes [1,2,3]. Ca^2+^ is stored in organelles such as vacuoles, mitochondria, and endoplasmic reticulum, where abiotic stresses such as salt, cold, and drought cause a rapid increase in Ca^2+^ concentration in the cytosol [3,4,5,6]. However, biotic stresses, pH dynamics, and phytohormones also can affect Ca^2+^ concentration [7,8,9,10]. In addition, pollen tube development and guard cell regulation are also associated with changes in Ca^2+^ concentration [8]. Calcium sensors or calcium-binding proteins recognize the modification in Ca^2+^ concentrations in plant cells, and downstream pathways are induced by affecting the phosphorylation status of calcium sensors and activating protein kinases [11,12]. Calmodulin (CaM), calcium-dependent protein kinases (CDPKs), and calcineurin B-like proteins (CBLs) are part of the known calcium sensors in plants [13]. CBLs are plant-specific sensors that, after sensing a specific calcium signature, can physically interact with a group of protein kinases, CBL-interacting protein kinases (CIPKs), to activate downstream signaling components [14,15,16]. CBL proteins share a common helix–loop–helix structural motif (the EF-hand composition), which acts as a Ca^2+^-binding region [17]. Moreover, it seems that the EF-hand composition could affect the affinity rate of calcium ions [17].

In the plant model system *Arabidopsis*, diverse roles were reported for CBLs: The *cbl1* mutant is very sensitive to abiotic stresses such as drought, extreme salinity, and hyperosmotic stress. Likewise, the *CBL9* gene is involved in ABA signal transduction and stress-induced ABA biosynthesis pathways [18]. In addition, it was reported that CBL9 and CBL1 participate in pollen germination and flower fertilization [19]. Furthermore, it was stated that CBL1 is involved in response to aluminum stress [20] and cold stress [21,22]. Moreover, CBL7 is associated with *Arabidopsis* responses to alkaline stress [23]. Interestingly, it was reported that CBLs, such as CBL3 and CBL4, could modulate the potassium channel and affect potassium homeostasis [21,24]. It has also been found that the expression patterns of the *CBL* genes are dependent on tissues and developmental stages and the type of stress. For example, *CBL1* expression is not affected by the external application of abscisic acid (ABA) but is induced in response to environmental stresses such as salt, cold, drought, and wounding [25]. While *CBL2* and *CBL3* do not respond to abiotic stress stimuli, they are transcriptionally induced by light stress [26]. *CIPK* genes also have differential expression patterns. For example, *CIPK9* transcriptional regulation is more induced in response to ABA treatment and is mainly activated in shoot tissues [27]. In addition, *CIPK* genes in *Medicago truncatula*, including *MtCIPK2*, *MtCIPK17*, and *MtCIPK18*, were found to be upregulated in response to salinity, PEG, and ABA treatments [28]. Recently, it has been reported that a *CIPK* gene from chrysanthemum, *CmCIPK8*, could affect the expression patterns of ion transport-related genes and may enhance tolerance to salinity [29]. Moreover, *CIPK10* in potatoes (*StCIPK10*) could increase tolerance to osmotic and drought stress by affecting the content of osmoregulation substances [30]. Additionally, it was reported that StCIPK10 can interact with several StCBLs, including StCBL4, StCBL8, StCBL1, StCBL6, StCBL12, and StCBL11 [30]. In *β vulgaris*, it was described that *BvCIPKs* are upregulated in response to NaCl treatment [31]. In *Saccharum spontaneum*, *CIPK* genes were shown to respond to abiotic stresses such as cold and water stress and ABA treatment [32]. Overall, it seems that cell signaling networks linked with CBL–CIPK play critical roles in response to abiotic stresses [33,34,35].

*A. littoralis* as a halophyte model can grow under high salt concentrations [36,37]. Identifying the genes related to tolerance in plants such as *A. littoralis*, a valuable germplasm, and determining their function can provide a better understanding of tolerance mechanisms in plants [38]. According to the mentioned materials above, the genes of CBL and CIPK families play a key role in responding to environmental stresses and regulating downstream signaling pathways, but these gene families have not been identified and investigated in *A. littoralis*. Here, we identified the members of CBL and CIPK families and analyzed their structure and evolution as well as their regulatory systems. In addition, the expression profiles of *AlCBL* and *AlCIPK* genes were evaluated under salinity in the root and leaf tissues of *A. littoralis*. 

## 2. Materials and Methods

### 2.1. Identification of CBL and CIPK Family Genes in A. littoralis

In this study, the putative protein sequences of CBL and CIPK in rice were retrieved from the RGAP database (http://rice.plantbiology.msu.edu/, accessed on 25 December 2022) and for *Arabidopsis thaliana* from the TAIR database (https://www.arabidopsis.org/, accessed on 25 December 2022). Sequences were used as queries in blastp and tblastn tools, E-value < 1 × 10^−10^, to identify members of *CBL* and *CIPK* gene families from the transcriptome platform e!DAL of *A. littoralis* [39]. The presence of PKinase and NAF domains in CIPK proteins, as well as EF-hand domains in CBL proteins, was tested and confirmed using the CDD database [40], SMART [41], and InterPro Scan [42]. The confirmed protein sequences were renamed based on their orthologs in *Arabidopsis*. Further AlCBL and AlCIPK proteins were analyzed using the ExPASy online database ProtParam tool [43] to predict their physiochemical properties, including molecular weight (MW), GRAVY, and isoelectric point (pI). 

### 2.2. Phylogenetic Analysis and Classification of AlCBL and AlCIPK Gene Families

To investigate the evolutionary relationships in calcium sensor gene families, the protein sequences of AlCBL and AlCIPK families, along with their orthologs in *Arabidopsis* and rice, were analyzed. First, the sequences were aligned using the ClustalW tool [44], and then a phylogenetic tree was drawn with the IQ tree software [45] using the maximum likelihood (ML) method with 1000 bootstrap replications. Finally, the tree file was restored and upgraded in the iTOL database [46].

### 2.3. Motif Analysis and Gene Structure of AlCBLs and AlCIPKs

Ten conserved motifs of AlCBL and AlCIPK protein sequences were predicted using the MEME motif finder [47] based on its default setting. In addition, the gene structure of *AlCBL* and *AlCIPK* genes was illustrated based on exon and intron distribution using TBtools [48].

### 2.4. Promoter Analysis of AlCBLs and AlCIPKs

In the current study, the upstream region, 1500 bp, of *AlCBL* and *AlCIPK* genes was analyzed using the PlantCARE tool (https://bioinformatics.psb.ugent.be/webtools/plantcare/html/, accessed on 25 December 2022) to identify the putative *cis*-regulatory elements.

### 2.5. Plant Materials’ Growth Conditions and Salt Treatments

The cultivation of *A. littoralis* seeds was carried out at a temperature of 25 ± 3 and a photoperiod of 16 h of light and 8 h of darkness. Then, the cloned samples were transferred to Hoagland’s solution, and after two months, salt stress treatment was started. In order to apply salinity stress, sodium chloride was gradually added; specifically, 100 mM salt was added to the solution every 3 days until the final concentration reached 600 mM. The sampling of leaf and root tissues was carried out in the time series of 0 (as a control sample), 3, 12, and 24 h after the application of salt stress. The collected samples were kept in a freezer at −80 for the next steps. All experiments were performed in three biological replications.

### 2.6. RNA Extraction and cDNA Synthesis

The extraction of total RNA from leaf and root tissues was carried out using a Trizol Kit (Threezol, Riragene). To remove genomic DNA from RNA, DNase I treatment (DNase I RNase-free, Thermo Scientific, Waltham, MA, USA) was applied. Finally, cDNA was synthesized using a RevertAid First-Strand cDNA Synthesis Kit (Thermo Scientific, Waltham, MA, USA) based on the company’s instructions and diluted four times.

### 2.7. Real-Time PCR

In the present study, the levels of mRNA abundance from six *AlCBL* and twelve *AlCIPK* genes were investigated in two tissues, roots and leaves, under salinity and normal conditions. Genes were selected based on phylogenetic analysis. The primers of candidate genes were designed using AlleleID [49] (Tables S1 and S2). The Maxima SYBR Green/ROX qPCR Master Mix (Thermo Scientific) was used to evaluate the relative expression based on the manufacturer’s instructions, with a Bio-Rad CFX96 machine. The temperature cycle was performed in two stages according to the manufacturer’s instructions: 10 min activation stage at 95 °C, 40 cycles at 95 °C for 15 s, and 60 °C for 1 min. In the current study, three reference genes, namely *AlUBQ*, *AlRPS3*, and *AlRPS3***,** were used for each tissue. The geometric mean of these genes was used to normalize the data. Finally, the relative expression levels of each target gene were calculated using the 2^−ΔΔCT^ method [50].

## 3. Results

### 3.1. Physicochemical Properties of AlCBLs and AlCIPKs

In the present study, six *AlCBL* genes and twenty *AlCIPK* genes were identified in the genome of *A. littoralis*. The evaluation of the physicochemical characteristics of CBL proteins revealed variable molecular weight in the range of 18.70 (AlCBL4.4) to 34.67 kDa (AlCBL10), and all AlCBLs were predicted as acidophilic proteins, pI less than 5.5 (Table 1). Furthermore, all AlCBLs (except for AlCBL10 protein) had negative GRAVY values, revealing that most AlCBLs have hydrophilic properties. In general, in terms of physicochemical characteristics, the AlCBL10 protein was different from other members of the AlCBL gene family, which can be more considered in molecular functional research. According to the genes’ physicochemical characteristics, AlCIPK family members showed more diversity than AlCBLs. Molecular weight in AlCIPKs ranged from 42.04 (AlCIPK10.6) to 58.97 kDa (AlCIPK10.1), and pI varied from 6.21 (AlCIPK21) to 9.28 (AlCIPK10.2).

### 3.2. Phylogenetic Analysis of AlCBLs

**Figure 1:**
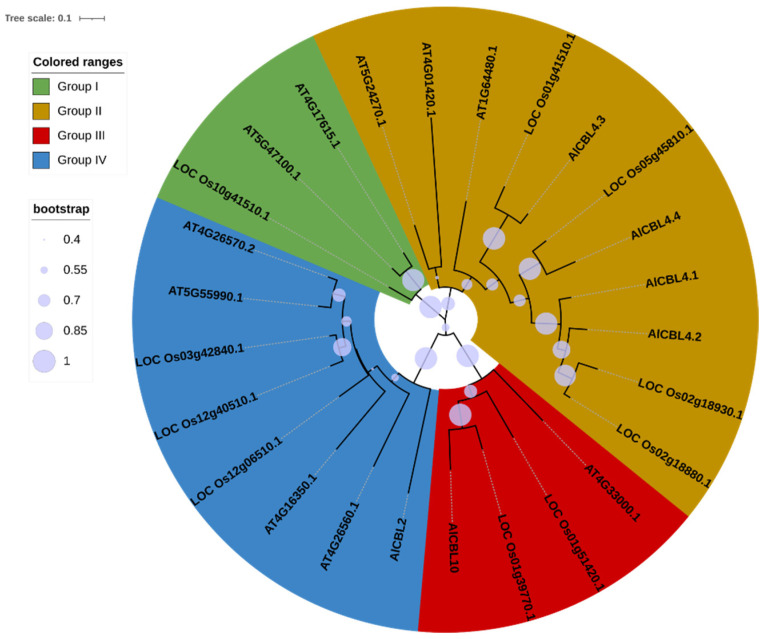
Phylogeny tree of CBL family proteins in *A. littoralis* (AlCBL), *Oryza sativa* (started with LOC Os), and *A. thalianas* (started with AT). The phylogenetic tree was drawn using the maximum likelihood (ML) method with 1000 bootstrap replications.

AlCBL proteins, along with their orthologs in rice and *Arabidopsis,* were subjected to phylogenetic analysis. The results disclosed that CBL proteins could be classified into four main groups (Figure 1). None of the AlCBLs could be identified in group I. AlLAC4.1, AlLAC4.2, AlLAC4.3, and AlLAC4.4 were located in group II, AlLAC10 in group III, and AlLAC2 in group IV. In addition, AlCBLs and rice CBLs showed more similarity to each other than *Arabidopsis* CBLs. Overall, our results revealed that the diversity in the CBL family occurred after the splitting of monocots and dicots.

### 3.3. Phylogenetic Analysis of AlCIPKs

**Figure 2:**
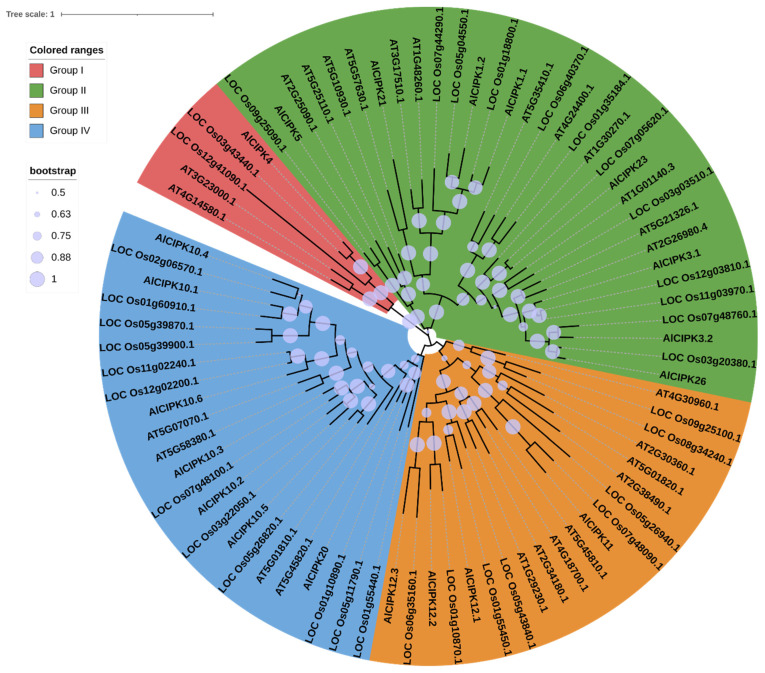
Phylogeny tree of CIPK family proteins in *A. littoralis* (AlCIPK), rice (started with LOC), and Arabidopsis (started with AT). The phylogenetic tree was drawn using the maximum likelihood (ML) method with 1000 bootstrap replications.

To determine the evolutionary origin of AlCIPKs, the phylogenetic tree of AlCIPKs with their orthologs in *Arabidopsis* (26 CIPK proteins) and rice (33 CIPK proteins) was drawn based on protein sequences (Figure 2). The results revealed that CIPKs could be separated into four main groups. The highest number of CIPKs was found in group III, and the lowest number was observed in group IV. Similar to AlCBLs, AlCIPKs also showed a close relationship with rice CIPKs. In addition, it can be concluded that the expansion of the CIPK family probably occurred after the derivation of monocots and dicots.

### 3.4. Gene Structure and Conserved Motifs of AlCBLs

**Figure 3:**
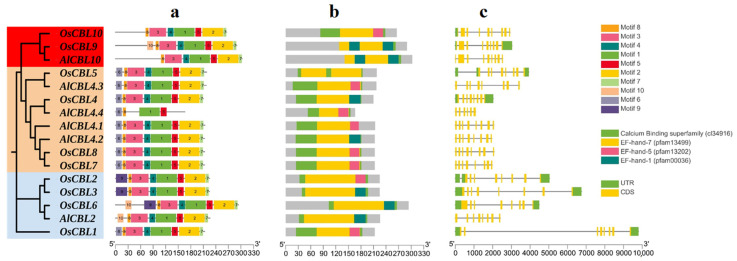
Structure analysis of AlCBL gene family. Distribution of conserved motifs (**a**), functional domains (**b**), and exon/intron (**c**) in AlCBL and OsCBL family members.

AlCBLs with their orthologs in rice (OsCBLs) were analyzed based on their conserved motifs and domain and gene structure (Figure 3). Ten conserved motifs were recognized in AlCBLs and OsCBLs; of those, motifs 6 and 9 were not detected in CBLs from group II, and motif 9 was only observed in OsCBLs from group III (Figure 3a). The calcium-binding superfamily, namely EF-hand 7, EF-hand 5, and EF-hand 1 domains, were observed in AlCBLs and OsCBLs, although they differed based on the location and number of domains (Figure 3b). In addition, two copies of EF-hand 7 and EF-hand 1 domains were found in AlCBL10 and its ortholog, OsCBL9, suggesting that AlCBL10 probably has more potential to interact with the downstream elements of involved pathways. In addition, *AlCBLs* were different based on their gene structure, and all *AlCBLs* had a high number of exons/introns (Figure 3c).

### 3.5. Gene Structure and Conserved Motifs of AlCIPKs

**Figure 4:**
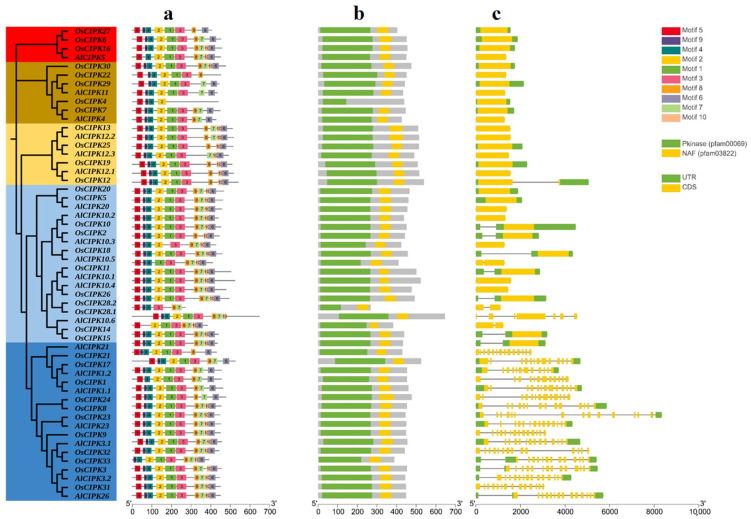
Structure analysis of AlCIPK gene family. Distribution of conserved motifs (**a**), functional domains (**b**), and exon/intron (**c**) in AlCIPK and OsCIPK family members.

To identify conserved motifs and determine the position of these motifs in KINAS and NAF domains, AlCIPK proteins with their orthologs in rice (OsCIPKs) were analyzed using the MEME tool (Figure 4a). The results show that the spatial distribution of the motifs in the investigated proteins is strongly conserved. All ten identified motifs were observed in AlCIPK3.1, AlCIPK3.2, AlCIPK10.1, AlCIPK10.2, AlCIPK10.4, AlCIPK20, AlCIPK23, and AlCIPK26 proteins, while motif 10 was not detectable in AlCIPK1.1, AlCIPK12.1, AlCIPK12.2, and AlCIPK12.3. Motif 5 was not detected in AlCIPK4, motif 4 was not detected in AlCIPK5, motif 1 was not observed in AlCIPK10.3, and motif 2 was not observed in AlCIPK10.5. In the AlCIPK1.2 protein, motifs 10 and 3, in the AlCIPK11 protein, motifs 10 and 6, in the AlCIPK10.6 protein, motifs 3 and 8, and in the AlCIPK21 protein, motifs 10 and 4 were not present. These results indicate that the main (conserved) motifs play an important role in the function of CIPK proteins. Moreover, two KINAS and NAF domains were identified in AlCIPKs and OsCIPKs (Figure 4b); all the studied proteins showed one copy of the KINAS and NAF domains. Based on gene structure analysis, 60% of *AlCIPK* genes have 1 exon and no intron, about 20% of genes have 14 exons and 13 introns, about 10% of genes have 2 exons and 1 intron, about 5% of genes have 13 exons and 12 introns, and about 5% of genes have 12 exons and 11 introns (Figure 4c).

### 3.6. Promoter Analysis

**Figure 5:**
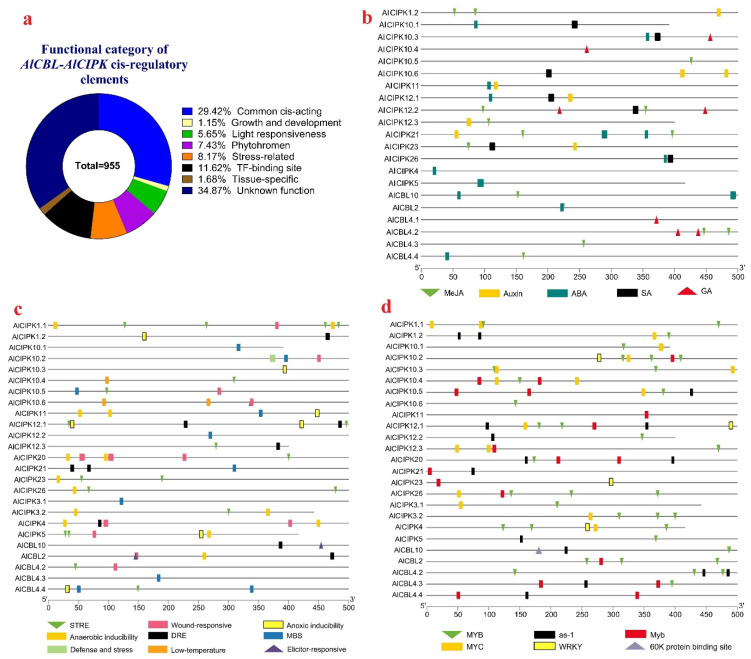
Distribution of *cis*-regulatory elements in the upstream of *AlCBLs* and *ALCIPKs.* Grouping of *cis*-regulatory elements based on their functions (**a**). Distribution of *cis*-regulatory elements involved in response to phytohormones (**b**), stress (**c**), and TF binding site (**d**). In this study, the upstream 1500 bp of *AlCBL* and *AlCIPK* genes was analyzed using PlantCARE.

In the present study, the upstream of *AlCIPKs* and *AlCBLs* was screened to identify the *cis*-regulatory elements related to stress, hormone, and growth and development (Figure 5). The most recognized elements were related to common *cis*-regulatory and elements with unknown functions (Figure 5a). In addition, putative *cis*-regulatory elements related to transcription factors’ binding site, response to phytohormones, and stresses were observed in the upstream sites of *AlCIPKs* and *AlCBLs*. The *cis*-regulatory elements involved in response to ABA hormone were frequently identified in the promoter sites of *AlCIPKs* and *AlCBLs* (Figure 5b). Moreover, the putative *cis*-regulatory elements related to GA, auxin, SA, and MeJA hormones were recognized in the upstream sites of *AlCIPKs*, while in *AlCBLs*, regulatory elements responding to GA and MeJA hormones were observed. The *cis*-regulatory elements involved in responsive to abiotic stresses, including low temperature, MBS, DRE, and STRE, and biotic stresses, including wound, elicitor, and defense mechanisms, were identified in the promoter regions of *AlCBLs* and *ALCIPKs* (Figure 5c). In addition, the binding sites of several TFs such as MYB, MYS, and WRKY were observed in the upstream sites of *AlCIPKs* and *AlCBLs* (Figure 5d). In general, *AlCIPKs* were richer than *AlCBLs* based on the number of stress-related *cis*-elements.

### 3.7. Expression Profiles of AlCBL Genes in Response to Salinity

**Figure 6:**
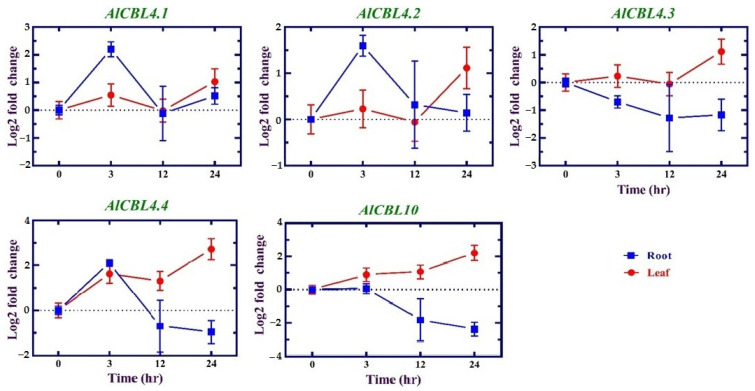
Expression patterns of *AlCBL* genes in response to salinity in two tissues: root and leaf. Expression levels are presented based on log2 fold change stress/normal condition.

The expression levels of *AlCBL* genes were investigated under salinity in root and leaf tissues. According to our results, *AlCBL2* was not expressed under the tested conditions. It seems that *AlCBL2* is not probably induced in response to salinity stress. After 3 h of salinity treatment, *AlCBL4.1*, *AlCBL4.2*, and *AlCBL4.4* showed an upregulation in root tissues (Figure 6). Three *AlCBL* genes, namely *AlCBL4.3*, *AlCBL4.4*, and *AlCBL10*, were differentially induced after 24 h; all three genes were upregulated in the leaf, while they were downregulated in the root. 

### 3.8. Expression Profile of AlCIPK Genes in Response to Salinity

**Figure 7:**
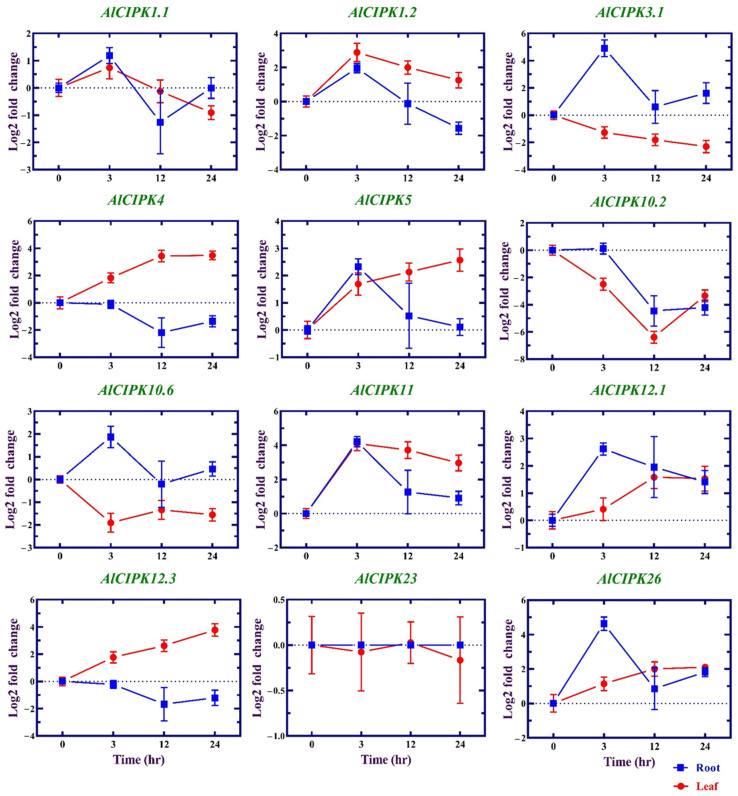
Expression patterns of *AlCIPK* genes in response to salinity in two tissues root and leaf. Expression levels are presented based on log2 fold change stress/normal condition.

In the root tissue, the expression levels of *AlCIPK1.2* (1.96 times), *AlCIPK3.1* (4.90 times), *AlCIPK5* (2.32 times), *AlCIPK11* (4.21 times), *AlCIPK12.1* (2.62 times), and *AlCIPK26* (4.63 times) were increased after three hours (h) of applying salt stress (Figure 7). In the leaf tissue, at 3 h after applying salt stress, *AlCIPK11* (4.10 times), *AlCIPK1.2* (2.89 times), *AlCIPK4* (1.82 times), *AlCIPK12.3* (1.77 times), *AlCIPK5* (1.69 times), *AlCIPK10.2* (−2.50 times), and *AlCIPK10.6* (−1.91 times) were more induced. In the root tissue, after 12 h of salinity, the *AlCIPK10.2* gene (−4.46 times) just showed a sharp downregulation, while in the leaf tissues, *AlCIPK4* (3.43 times), *AlCIPK10.2* (−6.39 times), *AlCIPK11* (3.72 times), *AlCIPK1.2* (2.00 times), *AlCIPK3.1* (−1.82 times), *AlCIPK5* (2.13 times), *AlCIPK10.6* (−1.34 times), *AlCIPK12.1* (1.58 times), *AlCIPK26* (2.00 times), and *AlCIPK12.3* (2.61 times) showed a significant modification in their expression levels after 12 hr. Interestingly, *AlCIPK10.2* was notably downregulated in both root and leaf tissues. In addition, the expression levels of *AlCIPK4* (3.47 times) and *AlCIPK12.3* (3.78 times) were increased in leaf tissue after 24 h. In total, *CIPKs* were more expressed in the leaf tissue, while *AlCIPK12.3* was expressed only in the leaf tissue, and *AlCIPK1.1* gene expression was observed only in the root tissue. *AlCIPK4, AlCIPK5, AlCIPK10.2*, *AlCIPK10.6, AlCIPK11*, and *AlCIPK12.3* genes were significantly expressed in the leaf tissue at all times of stress.

## 4. Discussion

Calcium sensors such as calcineurin B-like proteins (CBLs) and CBL-interacting protein kinases (CIPKs) not only participate in the processes of plant growth and development but are also involved in stress responses [30]. In the present study, the available genome of *A. littoralis* was used as a reference [38] and screened for the respective gene families. Six *AlCBL* genes and twenty *AlCIPK* genes were identified. Due to the importance of calcium-dependent signaling pathways, CBL and CIPK gene families have been studied in various plants. Notably, 23 *CBLs* and 58 *CIPK* genes were identified from the genome of *Medicago sativa* [28], 27 *CIPK* genes from potato [30], 9 *CBLs* and 30 *CIPK* genes from the pecan genome [51], 10 *CBLs* and 26 *CIPKs* from *Arabidopsis* [17], 7 *CBLs* and 20 *CIPK* genes from bread wheat [52], 7 *CBLs* and 23 *CIPK* genes from canola [53], 16 *CBLs* and 41 *CIPK* genes from quinoa [35], and 20 *CIPK* genes from sugar beet [31]. The different number of members of this gene family suggests that they may have been subjected to evolutionary pressures differently in each plant [54,55]. Based on their physicochemical properties, AlCBL proteins have similar properties, except for the AlCBL10 protein. The proteins of the AlCIPK family showed more diversity. This result supports the hypothesis that AlCIPKs are highly diverse due to their involvement in different pathways [56,57]. While *AlCIPKs* showed a high variation in terms of gene structure, *AlCIPKs* could be separated into two groups. This grouping was based on their low intron number (less than three introns) and high intron number (more than ten introns). Moreover, this feature has also been reported in previous studies, where *CIPKs* have been classified into two groups based on their intron structure [35,58]. It was stated that partial duplication has probably affected the intron number of gene family members [59]. Moreover, it was reported that the expression levels of genes can be affected by intron number, and genes with low intron number could be faster induced [60]. According to phylogenetic analysis, both AlCBL and AlCIPK families are closely related to their rice orthologs. This finding suggests that the diversity in these gene families occurred after the derivation of monocots and dicots species [61,62]. 

Halophyte plants have a high potential to grow in substrates with high salinity. Therefore, these species are of great interest to investigate the mechanisms of tolerance to salinity. Such mechanisms include the absorption, transport, and homeostasis of ions, osmotic regulation, and salt removal from leaves [37,38]. Although the cultivation of these plants is not an easy task, the germplasm of halophyte plants is considered a valuable source for providing genes resistant to environmental conditions, for the implementation of plant-breeding programs [63]. In the current study, the expression profiles of *AlCBLs* and *AlCIPKs* were investigated under salt stresses in the roots and leaves of *A. littoralis*. *AlCBLs* and *AlCIPKs* showed tissue-specific expression patterns. For instance, *AlCIPK* mRNAs were more in leaves than in roots, while *AlCBL4.3*, *AlCBL4.4*, and *AlCBL10* showed upregulation in roots and downregulation in shoots. This pattern might be related to the presence of as-1-specific motifs in the promoter region of *AlCBL* genes. Each of the *AlCBL4.3*, *AlCBL4.1*, and *AlCBL2* genes had two as-1 motifs, while six as-1 motifs were observed in *AlCBL4.2*, three as-1 motifs were observed in *AlCBL4.4,* and one as-1 motif was observed in *AlCBL10* promoter region. 

The results revealed that the co-expression pattern of *AlCBL* with *AlCIPK* was tissue-specific, and different co-expressions were observed in two tissues of leaves and roots. Based on the expression pattern, *AlCIPK3.1–AlCBL4.1* and *AlCIPK1.2–AlCBL4.4* genes can be potentially co-expressed in the root tissue, while in the leaf tissue, the *AlCBL10* gene can correlate with *AlCIPK5*, *AlCIPK12.3* and *AlCIPK26* genes. A positive correlation was reported between CBLs and CIPKs in response to stresses, such as salinity [64], drought [65], and disease [58]. In *Arabidopsis*, the interaction between CBL4 (called SOS3) and CIPK24 (called SOS2) could active the kinases and +/H+ antiporters called SOS1 and vacuolar H+-ATPase to increase stress tolerance [53,66,67]. Subsequent research in *Arabidopsis* showed that the AtCBL10 gene also interacts with AtCIPK24. Thus, the CBL10–CIPK24 complex interacts with vacuoles to protect the shoot from damage caused by salt stress [67]. This result suggests that calcium sensors may exhibit very different functions despite high sequence similarity or close evolutionary relationships. 

## 5. Conclusions

This review is the first comprehensive study of the family of calcium sensors with the aim of clarifying the evolution, expression patterns, and possible functions of the genes of this superfamily in *A. littoralis* in response to salinity stress. These findings provide information to predict the function of calcium sensor genes in plant tolerance to salinity stress. Additional studies on the expression of AlCBL and AlCIPK family genes under different abiotic stresses in future research can be useful in understanding the mechanism of gene expression adjustments related to the SOS pathway. The *AlCIPK* genes reported in this research, while providing preliminary information, provide a basis for identifying the functions and mechanisms of the stress response, especially the responses related to the CBL/CIPK pathway in the *A. littoralis* plant.

## Figures and Tables

**Table 1:** Physicochemical properties of identified AlCBL and AlCIPK encoded proteins from *A. littoralis*.

Family	Gene ID	Gene Name	Length (aa)	Intron Number	MW (kDa)	pI	GRAVY
**CBL**	Alg14121	*AlCBL2*	226	7	25.87	4.98	−0.219
	Alg15558	*AlCBL4.1*	214	7	24.35	4.71	−0.196
	Alg11525	*AlCBL4.2*	213	7	24.33	4.94	−0.259
	Alg8494	*AlCBL4.3*	217	7	24.88	5.19	−0.299
	Alg13204	*AlCBL4. 4*	166	5	18.70	4.78	−0.341
	Alg5886	*AlCBL10*	303	8	34.67	5.28	0.133
**CIPK**	Alg4127	*AlCIPK1.1*	473	12	53.48	6.52	0.372
	Alg7902	*AlCIPK1.2*	454	11	50.69	6.62	−0.320
	Alg7566	*AlCIPK3.1*	442	13	50.76	7.64	−0.460
	Alg12052	*ALCIPK3.2*	448	13	50.63	8.23	−0.407
	Alg15044	*AlCIPK4*	427	0	46.34	8.59	−0.115
	Alg5583	*AlCIPK5*	450	0	48.19	-	0.054
	Alg12300	*AlCIPK10.1*	523	0	58.97	9.03	0.401
	Alg9524	*ALCIPK10.2*	438	0	49.72	9.28	−0.260
	Alg4701	*AlCIPK10.3*	421	0	47.98	9.03	0.400
	Alg3308	*AlCIPK10.4*	478	0	54.66	9.13	0.514
	Alg13906	*AlCIPK10.5*	410	1	45.99	8.93	−0.307
	ALg9805	*AlCIPK10.6*	383	1	42.04	8.99	0.480
	Alg2698	*AlCIPK11*	433	0	47.40	8.95	−0.151
	Alg8115	*AlCIPK12.1*	516	0	57.36	8.64	−0.341
	Alg10559	*AlCIPK12.2*	515	0	57.47	8.06	−0.374
	Alg11449	*AlCIPK12.3*	490	0	54.06	8.84	−0.254
	Alg11347	*AlCIPK20*	456	0	51.64	9.08	−0.422
	Alg8711	*AlCIPK21*	430	13	48.54	6.21	−0.303
	Alg1003	*AlCIPK23*	449	13	50.51	9.16	−0.371
	Alg7179	*AllCIPK26*	448	13	50.44	8.41	−0.395

## Data Availability

Not applicable.

## Supplementary Materials

The following supporting information can be downloaded at: https://www.mdpi.com/article/10.3390/genes14030753/s1, Table S1: List of primers of *AlCBL* genes used in qPCR analysis; Table S2: List of primers of *AlCIPK* genes used in qPCR analysis.
