# Supplementary material for: Comprehensive Analysis of Calcium Sensor Families, CBL and CIPK, in Aeluropus littoralis and Their Expression Profile in Response to Salinity
Source: Genes (Basel). 2023 Mar 20;14(3):753. doi: 10.3390/genes14030753 (PMC10048465; doi:10.3390/genes14030753)
Supplement: Supplementary file 1 [file genes-14-00753-s001.zip › genes-2269109-supplementary.pdf]

Table S1. List of primers of *AlCBL* genes used in qPCR analysis

Gene symbol	Sequence (5'->3')	Primer length	Tm	GC%	Leaf PCR eff.		Amplicon	
					Mean %	SD	length	Tm
*ALCBL2**	**F:** CGGCATGATTAGCAAGGAAGAGT<br>**R:** GCGTCAGGATGGAAGACAGATAA	23<br>23	67.0<br>66.2	47.8<br>47.8	-	-	78.7	-
AlCBL4.1	**F:** CGAACGGTGACGGTAAGATAGA<br>**R:** GCTCCAGAGGCCAGAACAA	22<br>19	65.7<br>66.2	50.0<br>57.9	94.1	0.016	139	79.8
AlCBL4.2	**F:** CAAAGACGGGCTGATTCAC<br>**R:** AGGGTGGAAGATGTTGAGG	19<br>19	63.0<br>62.9	52.6<br>52.6	92.1	0.016	163	81.4
AlCBL4.3	**F:** TTATTGAACGGCATGAGCTAAAGG<br>**R:** ATTCCACTCCTCTTGATCTATCTTCC	24<br>26	65.6<br>65.6	41.7<br>42.3	92.9	0.013	155	78.3
AlCBL4.4	**F:** TCTAAGAGGGACAGGCTACATC<br>**R:** ACAGTGCTATCCGACAGACA	22<br>20	64.7<br>64.9	50.0<br>50.0	93.7	0.019	78.8	96
AlCBL10	**F:** GATGGATCTCCCTCAACTT<br>**R:** CGTTCACCGAGAAGCAT	19<br>17	60.0<br>61.1	47.4<br>52.9	92.4	0.029	69	81.3
AlUBQ	**F:** CTTGGTCTGCTGTTGTCTTG<br>**R:** CACGGTTCACTTATCCATCAC	20<br>21	63<br>63	50<br>48	89.8	0.025	200	80.5
AlRPS3	**F:** ATTCACTGGCTGACCGGATG<br>**R:** GTGCCAAGGGTTGTGAGGTC	20<br>20	63<br>63	55<br>60	93.6	0.022	107	78.5
AlEF1-a	**F:** TGCTGTCGGTGTCATCAA<br>**R:** CTTCCATCAAACGCCTCATT	18<br>20	63<br>62.5	50<br>45	95.2	0.016	97	80

* Not amplified

Table S2. List of primers of *AlCIPK* genes used in qPCR analysis

Gene symbol	Sequence (5'->3')	Primer length	Tm	GC%	Leaf PCR eff.		mplicon	
					Mean %	SD	length	Tm
AlCIPK1.1	**F:** GGCGTGTATCACAGAGA<br>**R:** AGGTGCGATGTAGTTAGG	17<br>18	59.6<br>59.9	52.9<br>50.0	94.5	0.030	156	81.3
AlCIPK1.2	**F:** TCTCTGAAGACGAAGGAAGGA<br>**R:** GGCTTGAGGTCCCTATGGTA	21<br>20	64.2<br>64.7	47.6<br>55.0	85.6	0.016	94	78.2
AlCIPK3.1	**F:** AGGGAACATTCGCAAAGG<br>**R:** CATCTTGTGCTTGAGAACCT	18<br>20	61.8<br>62.0	50.0<br>45.0	93.6	0.023	101	80.5
AlCIPK4	**F:** TCCTGTGGCGTCATCCT<br>**R:** ATGTTGGCGTCGTCGAAG	17<br>18	64.3<br>64.6	58.8<br>55.6	93.7	0.024	62	80.5
AlCIPK5	**F:** CCGCCCGTCATCATCAC<br>**R:** CGTCTTCCTCCGACCATC	17<br>18	64.4<br>63.4	64.7<br>61.1	89.2	0.018	61	80.7
AlCIPK10.2	**F:** ACACCGCCTCAACAATCATCAC<br>**R:** CAGCATTCACAGACATCACACCTT	22<br>24	67.6<br>67.6	50.0<br>45.8	90.0	0.024	138	77.8
AlCIPK10.6	**F:** CAGAGTCAAGGAGGCAAGATG<br>**R:** GACAACACCACAAGACCAGATG	21<br>22	64.6<br>65.5	52.4<br>50.0	92.8	0.024	128	80.4
AlCIPK11	**F:** GTGTAGTGAGGAGGGAAG<br>**R:** CTTGAAGACGGCGACTT	18<br>17	59.8<br>61.0	55.6<br>52.9	85.5	0.015	86	82.8
AlCIPK12.1	**F:** GCACAGCGTGATGGATG<br>**R:** AATGGTTGAGGAGCAGGAT	17<br>19	62.6<br>62.7	58.8<br>47.4	91.4	0.020	69	79.5
AlCIPK12.3	**F:** GTCCTCTTCGTGCTCGT<br>**R:** CGTTGTCCAGGTGGTTAC	17<br>18	62.8<br>61.8	58.8<br>55.6	92.3	0.029	55	80.2
AlCIPK23	**F:** GTCACGCCTTCGCTGTA<br>**R:** GCTTCGTCTCGTCACCTT	17<br>18	63.5<br>63.5	58.8<br>55.6	93.6	0.023	148	81.9
AlCIPK26	**F:** CCAATTATGTTGCTCCTGAGGTGAT<br>**R:** CTCCGCAAGACCAGACATCC	25<br>20	67.1<br>66.8	44.0<br>60.0	91.6	0.024	75	77.2
